# Demographic consequences of changing body size in a terrestrial salamander

**DOI:** 10.1002/ece3.6988

**Published:** 2020-12-13

**Authors:** Raisa Hernández‐Pacheco, Floriane Plard, Kristine L. Grayson, Ulrich K. Steiner

**Affiliations:** ^1^ Department of Biological Sciences California State University‐Long Beach Long Beach CA USA; ^2^ Department of Biology University of Richmond Richmond VA USA; ^3^ Swiss Ornithological Institute Sempach Switzerland; ^4^ UMR CNRS 5558 Biométrie et Biologie Evolutive University Claude Bernard Lyon 1 Villeurbanne France; ^5^ Evolutionary Biology Institut für Biologie Freie Universität Berlin Berlin Germany

**Keywords:** amphibian, body size, climate change, elasticity analysis, integral projection model, *Plethodon cinereus*

## Abstract

Changes in climate can alter individual body size, and the resulting shifts in reproduction and survival are expected to impact population dynamics and viability. However, appropriate methods to account for size‐dependent demographic changes are needed, especially in understudied yet threatened groups such as amphibians. We investigated individual‐ and population‐level demographic effects of changes in body size for a terrestrial salamander using capture–mark–recapture data. For our analysis, we implemented an integral projection model parameterized with capture–recapture likelihood estimates from a Bayesian framework. Our study combines survival and growth data from a single dataset to quantify the influence of size on survival while including different sources of uncertainty around these parameters, demonstrating how selective forces can be studied in populations with limited data and incomplete recaptures. We found a strong dependency of the population growth rate on changes in individual size, mediated by potential changes in selection on mean body size and on maximum body size. Our approach of simultaneous parameter estimation can be extended across taxa to identify eco‐evolutionary mechanisms acting on size‐specific vital rates, and thus shaping population dynamics and viability.

## Graphical abstract

Changes in climate can alter individual body size, and the resulting shifts in reproduction and survival are expected to impact population dynamics and viability. To quantify size‐dependent demographics, we used an integral projection model with capture–recapture likelihood estimates in a Bayesian framework. We used data from red‐backed salamanders and quantified a strong dependency of the population growth rate to changes in individual size mediated by potential changes in selection on mean body size and on maximum body size.

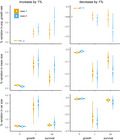

## 1 INTRODUCTION

Worldwide assessments predict that 15%–37% of terrestrial species will be at risk of extinction by 2050 (Thomas et al., 2004; Tilman et al., 2017). Several mechanisms of extinction predict associated changes in body size due to climate change (Baudron et al., 2014; Daufresne et al., 2009; Gardner et al., 2011, 2016; Sheridan & Bickford, 2011; Tseng et al., 2018). Yet, for most populations and species, we lack a quantitative understanding of the demographic consequences of changes in individual size and associated shift in the body size distribution within a population, even though the key components of fitness, survival and reproduction, are a function of body size (Honěk, 1993; Roff, 1986). To predict the dynamics and viability of natural populations, we need to understand the underlying demographic mechanisms shaping population growth rates through size‐dependent individual performance (Li et al., 2013; Rees & Ellner, 2009).

The association between body size and the demographic fates of individuals is driven by ecological and physiological processes, including resource availability, intra‐ or interspecific competition, and energy conversion efficiency. In particular, changes in environmental temperature affect metabolism and growth, which then regulates and feeds back into demographic fates (Lindmark et al., 2018; Savage et al., 2004). This relationship between size, demographic rates, and metabolism is particularly important in ectotherms, which compromise 99% of species worldwide and the vast majority of biomass (Atkinson & Sibly, 1997). At the projected rate of temperature increase under global warming (1.1–6.4°C by year 2100; IPCC, 2007), metabolic rates across a variety of ectotherm taxa are expected to increase by up to 75% (Bickford et al., 2010). This projected increase is expected to have a greater impact on the growth or survival of larger individuals through higher thermal and energetic requirements, and be most pronounced in habitats with scarce resources or where species are living near their thermal limits (Bickford et al., 2010). The resulting shifts toward smaller body sizes not only can affect population dynamics, but also could trigger cascading effects through food web alterations, as well as direct and indirect interactions in communities (Blaustein et al., 2010; Ohlberger, 2013; Reading, 2007).

Despite evidence for the role of size‐dependent individual performance in demography, quantifying the link between changes in size and population dynamics in the wild is limited. Advances in modeling populations structured by continuous traits provide the opportunity to quantify these dynamics (Elderd & Miller, 2016; Nicole et al., 2011; Ozgul et al., 2010). Changes in the distribution of individual sizes within populations can occur through changes in the selective pressure acting on either size‐specific survival, reproduction, or individual growth rates, or through phenotypic plasticity. The demographic consequences of these relative changes can be estimated using integral projection models (IPMs), describing the dynamics of populations structured by continuous traits (Easterling et al., 2000; Ellner et al., 2016). These population models can be extended to incorporate covariation between demographic rates, as well as their uncertainty, by including demographic parameters estimated in Bayesian frameworks (Elderd & Miller, 2016; Plard et al., 2019). IPMs built using parameters from Bayesian mark–recapture models, for example, provide measures of the impact (mean and uncertainty) from changes in selective gradients acting on natural population dynamics where not all individuals are perfectly tracked. However, this modeling approach has been limited by data availability and has rarely been applied to a single data source to simultaneously estimate survival and growth.

Here, we quantified the individual‐ and population‐level demographic effects of potential changes in selective pressures acting on body size in a population of the eastern red‐backed salamander, *Plethodon cinereus*. Specifically, we estimated the influence of body size on individual growth, survival, and reproductive success from a Bayesian capture–mark–recapture model and simulated the dependency of the population growth rate on these parameters by building an IPM. We determined uncertainty around all demographic parameters showing how selective forces can be studied in populations with limited data and incomplete recaptures. We estimated the elasticity of the population growth rate, the distribution of size, and the associated uncertainty to variation in survival and body size. This is important as several recent studies have reported *P. cinereus* body size changes and its potential for adaptive shifts in response to warming environments (Caruso et al., 2014; McCarthy et al., 2017; Riddell et al., 2018). Some populations have been found shrinking in size by up to 1.7% every decade (Caruso et al., 2014), while other populations are responding by increasing in size (McCarthy et al., 2017). These shifts in body size are expected to have important demographic consequences. The biological mechanism underlying the relationship of size and demographic rates in *P. cinereus* relies mostly on the ectothermic physiology and behavior of this species. Plethodontids are lungless salamanders that rely solely on cutaneous respiration for gas exchange and water uptake. Ova production declines as body size decreases (Fraser, 1980; Lotter, 1978), and future reproductive potential is negatively associated with past brooding status (Yurewicz & Wilbur, 2004). Thus, changes in body size directly affect their physiology through changes in surface‐to‐volume ratio, which translates into changes in fecundity and survival (Blaustein et al., 2010). Our findings demonstrate a strong dependency of the population growth rate on changes in individual size through changes in the selective pressure acting on mean body size and maximum body size, and suggest threats to the viability of terrestrial salamander populations under global warming.

## 2 METHODS

To analyze how changes in selective gradients acting on individual body size influence population dynamics, we built an integral projection model (IPM; Ellner & Rees, 2006). In IPMs, vital rates such as reproduction and survival are functions of one or several continuous traits (e.g., body size). Thus, IPMs allow studying the influence of variation in population trait distribution and variation in size‐vital rate functions on population growth rates by tracking individuals over time. We modeled the dynamics of snout‐to‐vent length (size) in the eastern red‐backed salamander to analyze the effect of changes in individual body size on demographic parameters at the population level. To describe the annual dynamics of size, we used three main functions that link demographic rates (survival, growth, reproduction) to size at each time interval (1‐year time‐step) during a 2‐year period. We parameterized these three main functions using parameters estimated from a Bayesian mark–recapture model.

In the sections below, we first describe the data used. Second, we describe the IPM we built and the associated functions (size‐dependent survival, growth in size, and size‐dependent reproduction). Third, we present how the parameters of our three main functions were estimated. Finally, we explain how we use this IPM and an elasticity analysis to investigate the influence of changes in selective gradients on population dynamics.

### 2.1 Studied population

**FIGURE 1:**
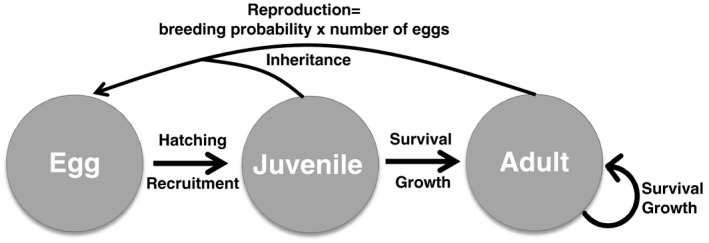
Life cycle of the red‐backed salamander used to build the IPM. Eggs develop directly into juveniles (~25 mm of snout‐to‐vent length). After their first year of age, individuals transition into adults. Sexual maturity is defined by size across both ages; immature individuals are <36 mm of snout‐to‐vent length, and mature individuals are ≥36 mm of snout‐to‐vent length

We conducted the study on a population of the eastern red‐backed salamander (*Plethodon cinereus*) located in the James River Park System, Richmond VA, USA (37°31′27 N, 77°28′29 W, elevation: 45 m). This area was used as a rock quarry in the early 1900s and now serves as a public park characterized by secondary mixed hardwood deciduous forest. This species of terrestrial salamander is particularly widespread and can be found as far north as Quebec and as far south as North Carolina (Petranka, 1998). Southern populations of red‐backed salamanders exhibit seasonal periods of surface activity separated by a long period of surface inactivity during the summer months where individuals retreat to underground refugia to decrease risk of desiccation (Hernández‐Pacheco et al., 2019; Nagel, 1977). This salamander is a dispersal‐limited species with narrow space use of <5 m and mean maximum movement distance ranging from 0.7 to 1.9 m (Hernández‐Pacheco et al., 2019; Muñoz et al., 2016; Sutherland et al., 2016). Leaf litter invertebrate dwellers compose their diet and eggs develop directly into juveniles with no aquatic stage (see life cycle; Figure 1).

### 2.2 Data collection

In November 2015, we established 5 × 10 m cover board arrays at three different sites (referred to as Sites 1, 2, and 3) in the study area, located at least 20 m apart from each other to avoid potential overlap of home ranges among individual salamanders. Each array consisted of 50 pine cover boards (30.5 × 30.5 × 2.1 cm) spaced 1 m apart in a rectangular grid (Sutherland et al., 2016). Each cover board served as an “open trap” that was surveyed on multiple capture occasions in two field seasons defined by availability for capture on the surface (season 1: autumn 2016–spring 2017; season 2: autumn 2017–spring 2018; Hernández‐Pacheco et al., 2019). During field season 1, we surveyed Sites 1, 2, and 3 on 6 capture occasions. During field season 2, we surveyed Sites 1, 2, and 3 on 7, 7, and 8 capture occasions, respectively. Capture occasions were separated by at least 3 weeks to maximize cover board effectiveness and decrease behavioral responses to disturbance (Hessed, 2012; Marsh & Goicochea, 2003). The field season ended once no more salamanders were observed on the surface in any of the three sites due to vertical migration to summer underground refugia (usually in May). Each capture occasion consisted of lifting each cover board and collecting all red‐backed salamanders underneath. Collected salamanders were transported to laboratory facilities at University of Richmond, where we identified them, measured their body size as snout‐to‐vent length, and sexed them when possible. On initial capture, individuals were given a unique mark by injecting a visual implant elastomer (Northwest Marine Technologies, Inc.) at up to four locations adjacent to each limb using combinations of up to five colors, a method found to be safe and reliable for individual identification in amphibians (Bailey, 2004; Grant, 2008). Eggs were counted by holding the live salamander against a light source and counting the visible eggs through the translucent skin. Given that external morphology cannot be used to determine the sex of juveniles (Gillette & Peterson, 2001) and because no significant sexual dimorphism in size has been found in southern populations of red‐backed salamanders (Leclair et al., 2006; Sayler, 1966), we assumed no sex differences in the influence of size on survival and growth. Within 24–48 hr of collection, individuals were released under the same board where they were collected. All procedures were approved by the Institutional Animal Care and Use Committee at the University of Richmond (Protocol 16‐03‐001). Research was conducted under scientific permits from the Virginia Department of Game and Inland Fisheries (nos. 056056 and 061682).

Across both field seasons, a total of 1,013 individuals were captured at the three cover board arrays (391, 260, and 362 in Sites 1, 2, and 3, respectively). Of these, 433 individuals were captured more than once (178, 97, and 158, in Sites 1, 2, and 3, respectively). For a single individual, the maximum number of detections was 8. Salamanders showed continuous surface activity from autumn to spring, retreating to underground refugia only during the summer months (June–August). Mean body size of the entire population was 38.17 mm (*SD* = 7.33) and 37.75 mm (*SD* = 7.54), during field seasons 1 and 2, respectively. Mean number of eggs was 8.34 (*SD* = 2.26) and 8.21 (*SD* = 2.01), during field seasons 1 and 2, respectively.

### 2.3 Population model

We parameterized an IPM to describe red‐backed salamander size‐dependent population dynamics and analyze the potential consequences of a change in selective pressure acting on size and impacting the population growth rate. The IPM describes the annual dynamics of the distribution of size (*x*) in the population. Between 2 years, individuals may survive and grow, and new juveniles may be produced (see life cycle; Figure 1). Thus, changes in the distribution of size (*x*) are the outcome of two main demographic processes: survival and growth ($P(x^{\prime }|x)$), and reproduction ($F(x^{\prime }|x)$), that is, recruitment of juveniles (individuals of 1 year of age) into the population. The population model described only the female portion of the population because the number of juveniles in the population depended on the number of eggs carried by females. Thus, male density and traits are assumed to have no effect on vital rates. Only body size structure was incorporated into the model as age was unknown for all individuals. Ignoring age structure should not influence our results because size correlates with age in this species. Moreover, the environmental impact on demography should influence all age classes with the same linear or nonlinear size‐dependent functions. We used a prebreeding model, and thus, the annual census time occurred while females were carrying eggs.

The density of female individuals in the population is described by the vectors **n**(*t*, *x*) and depended on time (*t*) and size. We used year as time interval. The density of females at *t* + 1 depends on the density of all female individuals at time *t* and is the sum of the number of surviving females and the number of juveniles they produce in the current year. In our annual prebreeding population model, juveniles are defined as individuals recruited in the population at 1 year of age:

$$n\left(t+1,x^{\prime }\right)=\int \left[P\left(x^{\prime }|x\right)+F\left(x^{\prime }|x\right)\right]n\left(t,x\right)dx.$$

The survival process included two functions. Females alive in year *t* can survive (survival function: **S**(*x*)) and grow (growth function: $G(x^{\prime }|x)$) to reach year *t* + 1. The reproductive process included five functions: the probability to produce eggs of a female of a given size (**P**(*x*)), the number of eggs produced by a reproducing female of a given size (**E**(*x*)), and the probability (**H**) that these eggs will hatch. Hatching females will recruit in the population at *t* + 1 with the probability **S^0^**. All juveniles then get a given size at *t* + 1 (juvenile size: **I**).

The density of females at *t* + 1 can be extended to:

$$n\left(t+1,x^{\prime }\right)=\int \left[G\left(x^{\prime }|x\right)S\left(x\right)+{\mathrm{IS}}^{0}\mathrm{HE}\left(x\right)P\left(x\right)\right]n\left(t,x\right)dx.$$

We developed two separate IPMs, one for each field season, that represented two different environments. These two IPMs have year as time interval and project the population from year *t* to year *t* + 1, but these years do not correspond to the field seasons. The first IPM assumed that annual survival rate and the number of eggs per female every year are the same as in field season 1. The second IPM assumed that annual survival and reproduction every year are the same as in field season 2. However, in both IPMs, growth was the same because it was estimated from field season 1 to field season 2.

#### 2.3.1 Survival and growth process

The survival function (**S**(*x*)) gives the number of surviving females from year *t* to year *t* + 1. We used a log link to model the survival probabilities as a function of a mortality hazard rate that depended on size at *t*. If females survive, they grow, and the growth function gives their size at *t* + 1 according to their size at *t*. We used a von Bertalanffy model as commonly done for this species to model the mean individual growth (Leclair et al., 2006; Muñoz et al., 2016). The parameters of this model were an individual growth rate and an asymptotic size. We modeled the growth function ($G(x^{\prime }|x)$) by a Gaussian density probability including a mean individual growth and a residual variance.

#### 2.3.2 Reproduction process

As no female smaller than 36 mm was recorded with eggs in our data, the probability of a female producing eggs (**P**(*x*)) was set to 1 if a female measured more than 36 mm, and 0 otherwise. Because some females larger than 36 mm may not produce eggs in some years or some females may not lay their eggs (Ng & Wilbur, 1995), our reproductive rate might be slightly overestimated (only 1 out of 226 females above this size was observed with no eggs when captured during one out of the 2 years sampled).

We modeled the number of eggs per female (**E**(*x*)) including the linear effect of female size. We fixed the hatching and recruiting probabilities to **H** = 0.9 and **S^0^** = 0.574, respectively, based on the limited information available in the literature (Homyack & Haas, 2009; Muñoz et al., 2016). We were not able to estimate these parameters from our data, but performed a sensitivity analysis to verify the robustness of our results to these assumptions (see Appendix S1B). We assumed an even sex ratio of juveniles.

The function giving the size of the juveniles (**I**) was modeled with a normal distribution with mean and variance size fixed to 25 and 2 mm, respectively, based on size distributions of juveniles from the literature (Leclair et al., 2006). This distribution was also reflected in our data as the smallest individuals were around 15 mm. Because we could not estimate any association between maternal size and offspring size, we used the conservative assumption that there is no link between female and offspring size, and thus, the distribution of juveniles does not depend on mother size. In this way, we assumed no plasticity prerecruitment and no genetic evolution. Including a high link (slope of 0.5) between maternal and offspring size did not change the main results (see Appendix S1B).

### 2.4 Estimation of the function parameters describing the demographic rates

We used a single model and a Bayesian framework to quantify the influence of size on survival, growth, and the number of eggs carried by females because all data came from the same capture–mark–recapture protocol during the 2 field seasons. Growth was estimated from field season 1 to field season 2 and thus did not depend on season. We estimated a monthly survival within each field season and explain below how it was then used to derive a yearly survival in the IPMs. We also estimated annual number of eggs per female for each field seasons. As individuals were captured in three different sites, we accounted for spatial heterogeneity by including a site effect on all parameters (Hernández‐Pacheco et al., 2019). However, we did not include movement among sites because this salamander is a dispersal‐limited species and plots are situated far apart to avoid migration (Hernández‐Pacheco et al., 2019).

#### 2.4.1 Size and growth

Individual growth was defined as the relationship between size (*X*_t_) in field season 1 and size (*X* *_t_*_+1_) in field season 2. As salamanders were not captured during all occasions of a given field season, we averaged individual seasonal size and estimated the average growth between the two field seasons (*N* = 650 individuals). Thus, we did not account for within‐season growth as our lack of data did not allow us to capture the nonlinear growth within field season. This assumption should not impact our results as the time interval of our population model is 1 year. The growth function was modeled using a von Bertalanffy model (Von Bertalanffy, 1957) as commonly done for this species (Leclair et al., 2006; Muñoz et al., 2016), usually defined as:

(1) $$X_{t}=L(1‐\exp\left(‐K\left(t‐t_{0}\right)\right)$$

where *K* is the individual growth rate in body size, and *L* is the asymptotic size. We allowed both values to depend on the three sites. Because we were interested in size growth between two successive seasons and we did not know the age of the individuals when captured, we directly used the growth increment form of the von Bertalanffy equation (Fabens, 1965):

(2) $$\begin{array}{cc} {\hat{X}}_{t+1} & =X_{t}+\left(L-X_{t}\right)\left(1-\exp\left(-K\right)\right)\\ & =\exp\left(-K\right)\left(X_{t}+L\left(\frac{1}{\exp\left(-K\right)}-1\right)\right) \end{array}$$

The likelihood of the size in field season 2 was the one of a normal density probability with a mean (${\hat{X}}_{t+1}$) equaled to the size predicted from the von Bertalanffy model (previous equation) and a variance ($\sigma_{X}^{2}$) modeled with a linear model:

(3) $$X∼N\left({\hat{X}}_{t+1},\sigma_{X}^{2}\right)$$

(4) $$\sigma_{X}^{2}=a+bX_{t}$$

where *a* and *b* are the site‐dependent intercepts and slopes of the linear model linking size in the first field season and the variance of the normal density probability function.

#### 2.4.2 Size and survival rate

We first estimated monthly average mortality hazard rate *H* (Ergon et al., 2018) to model the probability that individuals survive between each capture occasion. We used the likelihood of a Cormack–Jolly–Seber (CJS) model to analyze monthly apparent survival S_m_ and recapture *p* probabilities based on previous analysis of this population (Hernández‐Pacheco et al., 2019). We used a log‐log link to model monthly apparent survival and a logit link to model recapture probability. Because we expected large individuals to be more easily recaptured than small individuals, we also included an effect of size on the logit of recapture probability:

(5) $$\log\left(H\right)=e+fX$$

(6) $$\log\left(S_{m}\right)=‐H$$

(7) $$\text{logit}\left(p\right)=g+hX$$

where *e*, *f*, *g*, and *h* are the site‐ and season‐dependent intercepts and slopes linking observed size and survival and recapture probabilities. When individuals were not recaptured, size that was lacking has directly been simulated using the growth function within the same model (King et al., 2009).

We adapted survival probability to its true value between two capture occasions to the precision of 0.5 month because the time interval between capture occasions varied. For instance, time intervals between occasions 2 and 3 were 1.5 months in Site 1 and 2.5 months in Sites 2 and 3. Thus, survival between occasions 2 and 3 was $S_{m}^{1.5}$ and $S_{m}^{2.5}$, in Site 1 and Sites 2 and 3, respectively. In the population models (IPMs), annual survival was estimated as $S_{m}^{12}$.

#### 2.4.3 Size and number of eggs per female

We used a model including the linear effect of female size to predict the number of eggs carried by females. The likelihood of the number of eggs (Negg, *N* = 269) was the one of a normal density probability function with a mean ($\hat{N}egg$) estimated from a relationship including female size and a fixed estimated variance ($\sigma_{\mathrm{Negg}}^{2}$). We used a normal instead of a Poisson distribution because the former appropriately fit the distribution of the number of eggs per female (Appendix S1A).

(8) $$Negg∼N\left(\hat{N}egg,\sigma_{\mathrm{Negg}}^{2}\right)$$

(9) $$\hat{N}egg=c+dX$$

where *c* and *d* are the site‐ and season‐dependent intercepts and slopes of the linear model linking observed size and the predicted number of eggs carried by a female. We also tested a possible quadratic influence of size on the predicted number of eggs carried by a female by inspecting the 95% credible intervals of this parameter.

We performed this Bayesian analysis using JAGS (Plummer, 2003) run from R (R Core Team, 2019) using package jagsUI (Kellner, 2015). We defined normal distributions with mean 0 and variance 10^2^ for regression slopes and intercepts as vague priors (Kéry & Schaub, 2012). We generated 3 chains of length 100,000, used the first 50,000 as burn‐in, and sampled posterior values every 50 steps. Convergence of chains was assessed using the Gelman and Rubin convergence diagnostic (*R* < 1.01; Gelman & Rubin, 1992).

We built the different functions of the IPM based on the full Bayesian posterior distributions of all parameters obtained from the single Bayesian model. This allowed estimating uncertainty of population growth rate and associated elasticities. The IPMs were approximated as high dimensional discrete matrices (Easterling et al., 2000), and we used 100 size intervals from 0 to 65 mm. Program R (R Core Team, 2019) was used to build the IPM and to perform the associated elasticity analysis.

### 2.5 Analysis

We analyzed population dynamics at equilibrium. Thus, we estimated the asymptotic population growth rate from our IPMs, assuming individuals were distributed according to the stable size distribution. However, the actual influence of climate will vary each year with the transient distribution of size in the population. The mean and variance for juvenile and adult individual size in the real distribution were expected to vary slightly. But, the shape of the distribution is not expected to vary much as all individuals should be similarly impacted by climate.

To understand how changes in size could influence the population dynamics of red‐backed salamanders, we used our two IPMs and performed an elasticity analysis using manual perturbation analysis (instead of an analytical approach; Caswell, 2001). This relative change can occur either by a change in (i) the survival function through selection acting on size, (ii) the growth function through a change in individual growth rate, or (iii) the growth function through a change in asymptotic size. Thus, we estimated the elasticity of the population growth rate (*E_λ_*) in relation to (i) size–slope (*f*) of hazard rate, (ii) the individual growth rate (*K*), and (iii) the asymptotic body size (*L*). In practice, we estimated the relative change in the asymptotic population growth rate after a successive increase and decrease by 1% of these three parameters.

(10) $$E_{\lambda }=100\frac{\lambda_{p}‐\lambda_{0}}{\lambda_{0}}$$

where *λ*_0_ is the unperturbed population growth rate, and *λ*_p_ is the perturbed population growth rate obtained after a variation of 1% of the one of the parameter of the model.

## 3 RESULTS

### 3.1 Influence of size on demographic rates

**FIGURE 2:**
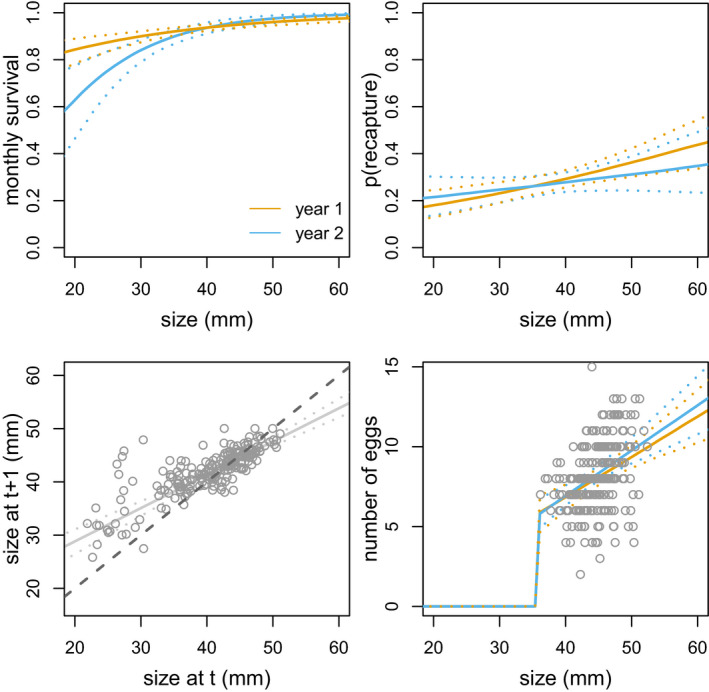
Influence of size (snout‐to‐vent length in mm) on growth, number of eggs, survival, and recapture rates. Field seasons 1 and 2 are presented with brown and blue lines, respectively. 95% credible intervals estimated are shown. For growth, the line of intercept 0 and slope 1 is shown with a dashed line

Between the two field seasons, individual growth rate was 0.480 [0.320; 0.642] (here and below, 95% credible intervals are given between square brackets). The asymptotic size was found to be 43.46 mm [42.16; 44.78] (Figure 2). Site did not have a large effect on individual growth. Indeed, 95% credible intervals for site effects on growth rate and asymptotic size included 0 (Table 1). The variance in individual growth was also not influenced by capture site but decreased with size such that small individuals had more heterogeneous growth from the first to the second season than large individuals.

**Table 1:** Posterior means with their 95% credible intervals and standard deviation of the parameters of the demographic rates of red‐backed salamanders

	Mean	*SD*	2.5%	97.5%
Growth (mean), von Bertalanffy model				
K	0.473	0.084	0.315	0.641
L	43.461	0.675	42.140	44.761
site2_K_	0.162	0.170	−0.162	0.516
site3_K_	0.161	0.136	−0.101	0.432
site2_L_	1.652	0.883	−0.151	3.270
site3_L_	−0.337	0.880	−2.098	1.382
Growth (variance), no link				
int	2.936	0.490	2.067	3.971
slp	−0.052	0.012	−0.078	−0.030
site2_int_	1.490	0.989	−0.318	3.468
site3_int_	0.204	0.672	−1.186	1.546
site2_slp_	−0.035	0.023	−0.080	0.008
site3_slp_	−0.006	0.017	−0.039	0.028
Number of eggs, no link				
int	−3.157	2.605	−8.333	1.974
slp	0.250	0.057	0.140	0.363
season2_int_	−1.292	3.496	−8.063	5.637
season2_slp_	0.034	0.078	−0.120	0.186
site2_int_	0.434	0.328	−0.201	1.077
site3_int_	−0.087	0.294	−0.667	0.472
Mortality rate, log link				
int	−0.817	0.361	−1.532	−0.145
slp	−0.048	0.009	−0.066	−0.029
season2_int_	2.001	0.726	0.498	3.385
season2_slp_	−0.050	0.020	−0.089	−0.011
site2_int_	0.674	0.158	0.375	0.975
site3_int_	0.395	0.130	0.152	0.663
Recapture probability, logit link				
int	−2.120	0.398	−2.929	−1.375
slp	0.031	0.010	0.012	0.051
season2_int_	0.472	0.613	−0.715	1.678
season2_slp_	−0.014	0.015	−0.044	0.015
site2_int_	−0.019	0.137	−0.287	0.245
site3_int_	0.097	0.111	−0.122	0.322

Note

Effects of season and sites on the different parameters are presented.

Abbreviations: int: intercept; slp: slope of the models.

Individual size positively influenced both reproduction and survival rates (Table 1, Figure 2) but had no quadratic effects on these demographic rates (quadratic effect of size on number of eggs: 0.002 [−0.007; 0.011] and monthly survival: 0.002 [−0.001; 0.004]). Females that measured between 43 and 47 mm (first and third quartile of the distribution of size among females carrying eggs) had 7.5 [6.9; 8.1] and 8.6 [8.0; 9.1] eggs, on average, respectively. Individuals measuring 25 and 45 mm (juvenile and older individual mean size in the population) had a monthly survival of 0.88 [0.84; 0.91] and 0.95 [0.94; 0.96], on average, respectively. Recapture probability was also influenced by individual sizes with smaller individuals having a lower probability of being recaptured (0.21 [0.16; 0.27] vs. 0.33 [0.29; 0.37], on average for 25 and 45 mm individuals, respectively). Sites and field seasons did not influence the number of eggs and recapture probability but influenced monthly survival (Table 1, Figure 2). Monthly survival was on average higher in the first season relative to the second season (Figure 2). Monthly survival was also higher in the Site 1 than in the two others (Table 1).

### 3.2 Influence of size on population dynamics

**FIGURE 3:**
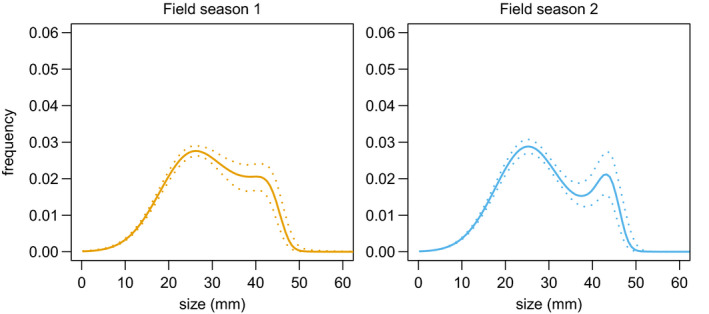
Predicted distribution of size (snout‐to‐vent length in mm) on growth from IPMs in the population of red‐backed salamander from Richmond, VA, USA. IPMs for field seasons 1 and 2 are presented with brown and blue lines, respectively

The proportion of juveniles predicted from the stable size distribution was higher in the IPM parameterized with survival and reproductive rates from the first field season relative to the IPM built for the second field season (Figure 3). A population modeled with demographic rates from the first season was characterized by higher survival of small individuals and thus a higher number of individuals reaching the reproductive stage than a population modeled by the demographic rates of season 2. Therefore, a higher population growth rate (*λ* = 0.801[0.682; 0.924]) was obtained using demographic rates of season 1 compared with those of season 2 (0.731 [0.576; 0.892]).

**FIGURE 4:**
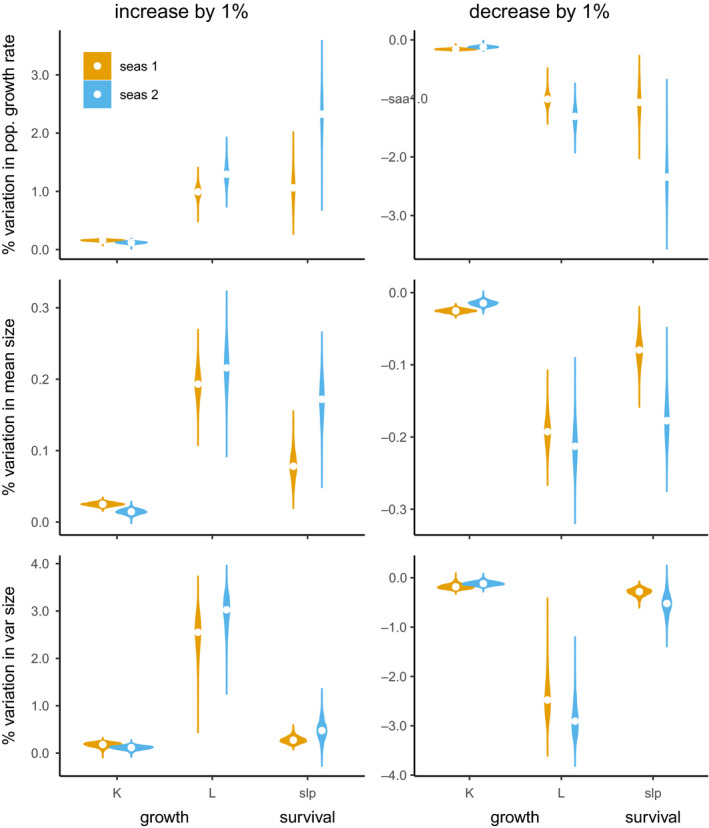
Elasticity analysis. Proportional changes in population growth rate, and in mean and variance of the stable size distribution after a 1% increase or a 1% decrease of the two parameters of the growth functions: the growth rate *K*, the asymptotic snout‐to‐vent length *L*, and the slope of the survival function (slp). Results from IPM parameterized from field seasons 1 and 2 are presented in brown and blue, respectively. These violin plots show the density probability for elasticities based on the full posterior distribution of the Bayesian model

The elasticity analysis revealed that changes in the slope of the survival function (selection in size) or in the asymptotic size can have major impacts on the distribution of size in the population and, thus, on population growth rate (Figure 4). A 1% decrease in the slope of the survival function with respect to size decreased the population growth rate by 1.7% on average (1% and 2.5% in population models using demographic rates of field seasons 1 and 2). A decrease in the asymptotic size by 1% (about: 0.5 mm) decreased the population growth rate by 1.2%, on average. A decrease in the individual size growth rate had a relatively smaller impact (1% decrease in individual size growth decreases *λ* by 0.2%). A 1% increase in these three parameters had the same but opposite effects on the population growth rate (Figure 4).

## 4 DISCUSSION

Our Bayesian mark–recapture model allowed us to estimate the parameters of the growth function and influence of size on survival simultaneously, generating estimates of the uncertainty around all linked parameters. In this way, we show the explicit link between population demography and size structure dynamics in the eastern red‐backed salamander. Along with other plethodontids, this species has a high sensitivity to temperature warming (Moore et al., 2018; Riddell et al., 2018), and has been hypothesized to undergo phenotypic changes under climate change. These changes include some natural populations decreasing in size (Caruso et al., 2014), while others increasing in size (McCarthy et al., 2017). Our study suggests that these shifts could lead to significant changes in the viability of our study population, a response potentially expected for other terrestrial ectotherm populations.

We studied the dynamics of a red‐backed salamander population over two field seasons, and our analysis supports that body size can influence demographic parameters through several mechanisms, including rapid growth during juvenile stages (Biddle et al., 2017; Leclair et al., 2006; Nagel, 1977) and positive associations between female fecundity and body size (Leclair et al., 2008; Nagel, 1977; Yurewicz & Wilbur, 2004). As found in many ectotherms, we also show that larger body sizes have higher survival in our study population. We decomposed these mechanisms using a quantitative model and addressed the influence of individual size dynamics on demographic parameters at the population level.

Rather than accounting explicitly for plasticity in body size as a response to temperature or other environmental variation (i.e., no environmental variables were included in our analysis), we addressed generally the relative influence of potential changes in selective pressures through survival or variation in growth on population demography using an elasticity analysis. We did this by focusing on reported changes in body size. For example, populations of red‐backed salamanders have been found to be decreasing in size at the rate of 1.7% per decade, potentially due to warmer environments (Caruso et al., 2014). These reductions in body size at the population scale are expected to occur through mechanisms that either decrease individual body sizes or increase the proportion of juveniles, both mediated by differential survival or growth (Caruso et al., 2014). Our prospective analysis based on a 1% change in either the survival and growth functions revealed that potential changes in survival and maximum (asymptotic) growth, rather than changes in individual growth rate, would have a greater influence on the population growth rate in the future. Therefore, if warming affects survival or maximum growth potential, then we can expect changes in the population growth rate due to climate change. It is important to note that the real impact of growth and survival rates on population growth rate will depend on the actual influence of climate change on these rates. Our elasticity analyses showed that a 1% decrease in survival rate or maximum growth would lead to a negative shift in the size distribution of surviving individuals. In this scenario, the size distribution of salamanders is smaller and thus they survive and reproduce less, resulting in a decreased population growth rate by a notable 1% to 2% annually. Yet, a 1% variation in survival rate would result in only 0.2% change in mean size in our salamander population, which might appear negligible relative to other *P. cinereus* populations. At the opposite end, 1% increase in the survival slope would result in an increased population growth rate of 1% to 2% annually.

Our analysis suggests that eco‐evolutionary mechanisms acting on size‐specific survival and maximum growth are major drivers of population viability. By 2100, global temperatures are expected to increase by 1.1–6.4°C (IPCC, 2007; Peters et al., 2012) and the resulting metabolic demands are expected to induce up to a 53% decrease in body size in several amphibian populations (Bickford et al., 2010). As reported by Caruso et al. (2014), a 7% reduction in body size in our current population (mean size: 38.0 mm) would result in a mean size of 35.3 mm, which is an approximate size for sexual maturity in *P. cinereus*. On the other hand, a 53% reduction in body size would result in a mean size of 17.9 mm, a size not viable for population growth. Furthermore, females can also impact offspring body size through postoviposition maternal effects, where a positive correlation has been found between size at hatching and female size (Crespi & Lessig, 2004). As we did not account for this maternal effect in our model, the potential predicted effects of changes in size on population growth rate from our analysis may be conservative.

Several recent studies have focused on the quantification of body size changes and the potential for adaptive shifts in salamander size in response to climate change (Caruso et al., 2014; McCarthy et al., 2017; Riddell et al., 2018). Contradictory findings on the direction of the change in body size among *P. cinereus* populations have been reported (Li et al., 2013). These contradictions center around detection and whether sampled individuals are representative of the true population and changes over time (Adams & Church, 2008; Connette et al., 2015; Grant, 2015). The controversy over declining body size impacts future conservation plans beyond salamanders and amphibians. In birds, reduced body size has been associated with increases in the surface‐to‐volume ratio and therefore improved metabolic rates through conduction in warmer environments (Gardner et al., 2009; Yom‐Tov, 2001). However, these negative shifts in size may feedback into decreased survival as a consequence of body mass reductions (Gardner et al., 2016; McKechnie & Wolf, 2012). In mammals, many populations have experienced increases in size instead due to increased energy savings and food availability with increasing temperatures (Meiri et al., 2009; Proffitt et al., 2007; Yom‐Tov & Yom‐Tov, 2004, 2005). However, decreased size in younger stages as a response to increased energetic demands has also been reported, suggesting reduced reproductive performance of adults and juvenile survival (Rode et al., 2010). The core of the issue is that powerful size‐structured population models need longer time series of individual data to get accurate estimates of plastic and evolutive response and to inform future management of wild populations (Clutton‐Brock & Sheldon, 2010).

Our modeling approach using eastern red‐backed salamanders quantifies the linkage between size‐dependent individual performance and population dynamics, demonstrating a strong dependency of the population growth rate to potential changes in individual size distribution. Specifically, our approach accounts for observed size‐specific survival, growth, reproductive success, and detectability of individuals in order to quantify the sensitivity of the population growth rate to changes in size and determine viability. These size‐dependent and temperature‐regulated demographic alterations are especially important in ectothermic species where the link between daily activities and size is well established.

## CONFLICT OF INTEREST

We declare no conflict of interest.

## AUTHOR CONTRIBUTIONS

**Raisa Hernández‐Pacheco:** Conceptualization (equal); data curation (supporting); funding acquisition (equal); investigation (equal); methodology (equal); project administration (equal); supervision (equal); visualization (equal); writing—original draft (lead); writing—review and editing (equal). **Floriane Plard:** Formal analysis (lead); methodology (equal); validation (equal); writing—review and editing (equal). **Kristine Grayson:** Data curation (lead); funding acquisition (equal); investigation (supporting); resources (lead); writing—review and editing (equal). **Ulrich K Steiner:** Conceptualization (equal); methodology (equal); project administration (equal); supervision (equal); validation (supporting); writing—review and editing (equal).

## Supporting information

Appendix S1

## Data Availability

All data and R codes are available in Dryad https://doi.org/10.5061/dryad.r7sqv9s9r
