# Supplementary material for: Demographic consequences of changing body size in a terrestrial salamander
Source: Ecol Evol. 2020 Dec 13;11(1):174–85. doi: 10.1002/ece3.6988 (PMC7790640; doi:10.1002/ece3.6988)
Supplement: Supplementary file 1 — Appendix S1 [file ECE3-11-174-s001.pdf]

Supplementary material

Appendix A: Supplementary figure

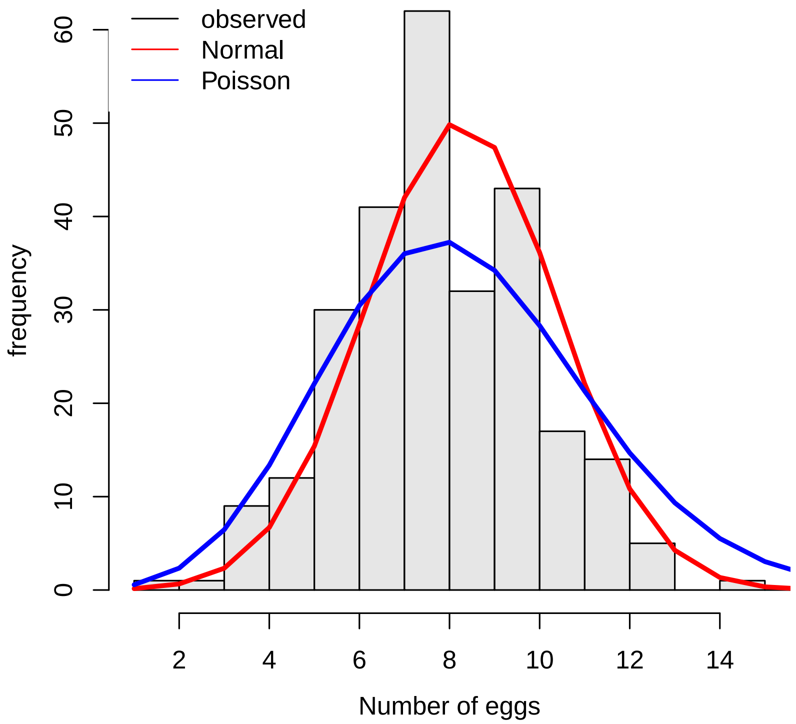

Figure A1: Observed distribution of the number of eggs carried by red-backed salamander females. Theoretical Normal (red) and Poisson (blue) distribution are also shown

Appendix B: Sensitivity Analysis

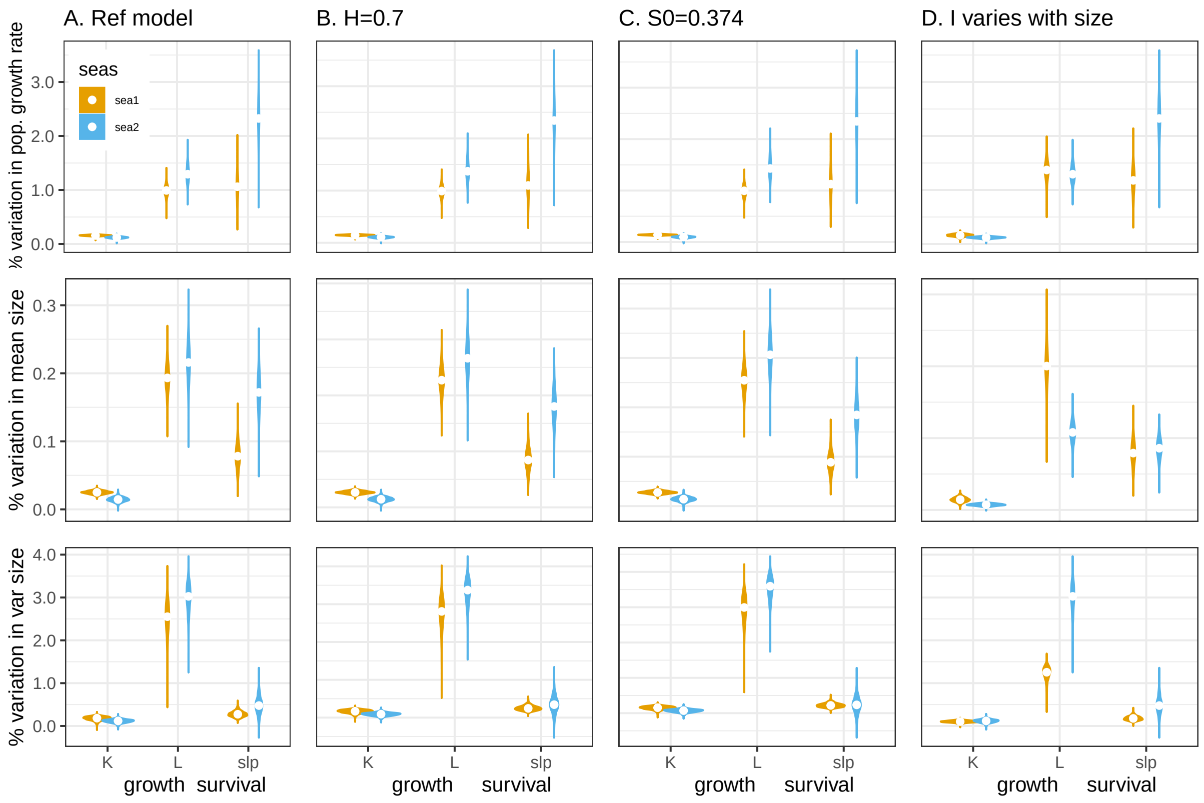

Figure B1: Sensitivity analysis. We plot the same figure as Figure 4 representing the elasticity analysis for 4 different models: A: Reference model used in the main analysis including hatching and recruiting probabilities of $\mathbf{H} = 0.9$ and $\mathbf{S^0} = 0.574$, respectively and a fixed mean size of the juveniles ($\mathbf{I} = N(25, 2)$), B: same as reference model except $\mathbf{H} = 0.7$, C: same as referent model except $\mathbf{S^0} = 0.374$, D: same as referent model except size of juveniles depended on maternal size $\mathbf{I} = N(2.5 + 0.5X_t, 2)$, where X_t is maternal mass at reproductive age. We compared the sensitivity of our results to variation in hatching and recruiting probabilities and to the form of the inheritance function by comparing under these four models: proportional changes in population growth rate, and in mean and variance of the stable SVL distribution after a 1% increase of the two parameters of the growth functions: the growth rate K and the asymptotic SVL L and of the slope of the survival function (slp). Field seasons 1 and 2 are presented with light and dark grey lines, respectively..
